# Current state of antimicrobial stewardship and organ transplantation in Spain

**DOI:** 10.1111/tid.13851

**Published:** 2022-10-18

**Authors:** Jose Tiago Silva, José María Aguado

**Affiliations:** ^1^ Unit of Infectious Diseases University Hospital “12 de Octubre” Instituto de Investigación Hospital “12 de Octubre” (i+12) Centro de Investigación Biomédica en Red de Enfermedades Infecciosas (CIBERINFEC) Madrid Spain; ^2^ Departamento de Medicina Universidad Complutense Madrid Spain

**Keywords:** antibiotic, antimicrobial stewardship, prophylactic therapy, solid‐organ transplantation

## Abstract

**Introduction:**

Solid‐organ transplantation (SOT) remains the best therapeutic option for end‐stage organ disease. Regrettably, SOT recipients are disproportionately affected by nosocomial infections produced by multidrug‐resistant (MDR) microorganisms and antimicrobial adverse events. Both have a negative impact on the patient´s outcome.

**Methods:**

Description of data concerning the antimicrobial stewardship program (ASP) in SOT recipients of the University Hospital “12 de Octubre”, and review of other Spanish ASPs.

**Results:**

From May 2017 to December 2021, the ASP issued 2.785 recommendations. Approximately, 4.9% were aimed at improving the antimicrobial treatment administered to SOT recipients. Treatment discontinuation or change to a better therapeutic regimen was recommended in 51.8% and 26.3% of cases, respectively. The acceptance rate of the recommendations was close to 92%. Between June 2015 and March 2016, a quasi‐experimental study consisting of a joint ASP and hospital‐acquired infection control (HAIC) initiative, which included kidney transplant recipients, reported a significant reduction in the consumption of meropenem, vancomycin and ciprofloxacin, and a reduction in the incidence of global bacterial infections, upper urinary tract infections, and cystitis. Although Spain has several robust regional ASPs (e.g., VINCat and PIRASOA), data specifically concerning SOT patients is lacking.

**Conclusion:**

ASP coupled with HAIC programs have proven to be effective in SOT, and should be implemented in centers that perform transplantation. Since data is scarce, Spanish centers which have ASP should report their experience in SOT. Understanding the efficacy and safety of the Spanish ASP´s intervention in the SOT population is essential and deserves further study.

## 1 INTRODUCTION

Spain is the European country with the highest rates of organ donation and transplantation procedures.^1^, ^2^ Despite the advances in surgical techniques and in infection control and prophylaxis, bacteria remain the most important cause of infection after solid organ transplantation (SOT),^3^ with a significant impact on allograft function, and overall morbidity and mortality. Moreover, in the past decades, there has been a steady increase in the number of bacterial infections produced by vancomycin‐resistant enterococci,^4^ and multidrug‐resistant (MDR) and extensively‐drug‐resistant (XDR) *Enterobacteriaceae* and non‐fermenting gram‐negative bacteria (GNB).^5^, ^6^, ^7^ Antibiotics active against these bacteria have several limitations, such as a lack of data on efficacy and outcomes in SOT recipients, a lesser‐known rate of adverse events, and, especially important, most are only available for parenteral administration, making it impossible for a swift hospital discharge.^8^ Finally, the overuse of antibiotics is responsible for the increasing trends of *Clostridioides difficile* infection (CDI) in SOT recipients, which is associated with more frequent recurrences, graft loss, and mortality.^9^

Equally worrisome is the increased rate in Spain of fluconazole non‐susceptible *Candida* species^10^ and azole‐resistant *Aspergillus fumigatus*.^11^ Ganciclovir‐resistant cytomegalovirus after SOT is also an emerging problem.^12^ Second‐line and third‐line drugs required to treat these microorganisms are associated with higher rates of side effects.

Prevention of infectious complications after SOT requires correct prophylactic therapy immediately before and after surgery.^13^ Additionally, in order to mitigate the spread of MDR/XDR strains, it is mandatory to implement measures such as hand hygiene, contact isolation, or selective bowel decontamination. Nonetheless, the key element in order to reduce the rates of MDR/XDR microorganisms and antimicrobial adverse events is an adequate antimicrobial policy, which optimizes the drug´s selection, timing, dosing, duration, and route of administration. The latter can only be achieved through a proficient and robust antimicrobial stewardship program (ASP).^13^

Over the past decade, there has been an effort in implementing ASP in the majority of the Spanish medical centers.^14^ The primary objectives are to improve the patient´s clinical outcomes, reduce the rate of adverse events related to the use of antimicrobials, including bacterial resistance, and ensure cost‐effective therapies. Recommendations vary from collecting microbiological samples before starting antibiotic treatments, to the optimization of antimicrobial treatment.

In this issue, we will be reviewing the data concerning ASPs in SOT, and specifically, how SOT Spanish programs can benefit from ASP.

### 1.1 Importance of ASP in SOT

Most of the empiric and targeted antimicrobial treatment prescribed to SOT recipients, as well as its duration, solely depends on the attending physician's decision. This can be explained by the lack of guidelines and recommendations by expert groups and infectious diseases societies concerning the use of antimicrobials in this specific population and by the diagnostic uncertainty and elevated risk of infections produced by MDR and XDR that characterizes most cases.^15^ Therefore, in recent years, there has been a call for ASP in SOT as a means to optimize antimicrobial prescribing and avoid many of its negative consequences, ultimately improving the patient´s outcome.^16^, ^17^

Recent studies have shown that ASP in SOT not only has a positive impact but that its implementation is feasible.^18^ A quasi‐experimental retrospective study conducted in a Canadian tertiary‐care teaching institution from January 2010 to December 2014 determined the impact of 5‐year serial infection control and ASP intervention on surgical site infections (SSI) among patients undergoing hepatobiliary surgery and liver, kidney transplantation (KT), pancreas, and simultaneous pancreas‐KT.^19^ Outcomes were compared between a preintervention group (2010–2011) and a postintervention group (2012–2014). After the implementation of the interventions, a decrease of 51.6% in SSI rates among SOT recipients was observed between the pre‐ and the postintervention groups (odds ratio [OR], 2.2; 95% confidence interval [CI], 1.4–3.5; *p* = 0.001). Moreover, the ASP intervention increased the overall conformity to the recommended surgical prophylaxis protocol by 15.2% (95% CI, 5.4–24.9; *p* < 0.003). An ASP in an Italian SOT center, which optimized antifungal use through clinical interventions in recipients diagnosed with infectious fungal infections, reported a better use of antifungal drugs in terms of appropriateness and consumption, with stable clinical and microbiological outcomes.^20^

### 1.2 ASP and SOT in Spain

One of the first Spanish hospitals to have an ASP was the University Hospital “12 de Octubre” (HU12O), a 1300‐bed university hospital located in Madrid. The non‐compulsory program for the assessment and control of antibiotic treatment (PACTA, by its acronym in Spanish) was implemented in March 2002 and was initially performed in six different departments of the hospital by a team of Infectious Diseases specialists.^21^ In 2008, this non‐compulsory program also began monitoring the antifungal therapy administered to adult patients,^22^ and by 2018 was being performed on a daily basis in almost all departments of the HU12O. Every day, a dedicated Infectious Diseases specialist monitors and optimizes all antimicrobial treatments that include broad‐spectrum antibiotics, such as carbapenems, active antibiotics against methicillin‐resistant *Staphylococcus aureus* or carbapenemase‐producing GNB and different antifungal drugs, including azoles, echinocandins and amphotericin B (Table 1). All cases are discussed personally with the treating physician before deciding on the recommendations. From May 2017 to December 2021, the PACTA issued 2.785 recommendations. Interestingly, 137 (4.9% of all given recommendations) were aimed at improving the antimicrobial treatment administered to SOT recipients (Table 2). Most patients were lung and KT recipients (68 and 51 patients, respectively). It was recommended to discontinue the antimicrobial treatment in almost half of the cases (51.8%), while in 26.3% the antimicrobial therapy was switched to a better therapeutic regimen. It is important to mention that the acceptance rate of the program´s recommendations was close to 92%, proving that the program was always welcomed by the clinicians who attend SOT patients. It is also noteworthy to refer that, in an effort to optimize the use of antimicrobials and reduce the incidence of bacterial infection among hospitalized KT recipients, between June 2015 and March 2016, we conducted a quasi‐experimental study consisting of a joint ASP and a hospital‐acquired infection control (HAIC) initiative.^23^ During this period, an Infectious Diseases specialist reviewed and optimized the antimicrobial treatments of all KT recipients admitted to the hospital, while an intensive HAIC program, which reinforced actions directed at reducing the in‐hospital spread of MDR microorganisms, was concurrently implemented. A historical cohort of KT recipients during the immediately preceding period (from June 2014 to March 2015) was used as a comparator. A total of 200 KT recipients were included (100 recipients in each cohort, respectively). The study reported a reduction in the consumption of meropenem (incidence rate ratio [IRR], 0.61; 95% CI, 0.51–0.72; *p* < 0.001), vancomycin (IRR, 0.62; 95% CI, 0.5–0.77; *p* < 0.001) and ciprofloxacin (IRR, 0.64; 95% CI, 0.52–0.78; *p* < 0.001). There was also a reduction in the incidence of global bacterial infections (IRR, 0.53; 95% CI, 0.35–0.82; *p* = 0.002) and, specifically, upper urinary tract infections (IRR, 0.51; 95% CI, 0.28–0.90; *p* = 0.01) and cystitis (IRR, 0.37; 95% CI, 0.15–0.83; *p* = 0.01). A non‐significant trend for a lower incidence of infections due to extended‐spectrum beta‐lactamase‐producing *Enterobacteriaceae* was also described (IRR, 0.53; CI 95%, 0.20–1.34; *p* = 0.15). The authors concluded that a joint approach based on ASP and HAIC was effective in improving the use of antimicrobials and in reducing the incidence of bacterial infection among KT recipients.

**TABLE 1:** Antimicrobials monitored by the antimicrobial stewardship program implemented in the University Hospital “12 de Octubre”

**Antimicrobials** ^a^, ^b^
Antibiotics Carbapenems (meropenem and imipenem) Ceftazidime‐avibactam Ceftolozane‐tazobactam Daptomycin Linezolid Tedizolid Tigecycline Ceftaroline Fosamil Fluoroquinolones (ciprofloxacin, levofloxacin, and moxifloxacin)^c^ Metronidazole^c^
Antifungals Azoles (fluconazole^c^, voriconazole, posaconazole, and isavuconazole) Liposomal amphotericin B Echinocandins (caspofungin, micafungin, and anidulafungin)

^a^ Antiviral drugs are not monitored.

^b^ Antibiotics are normally revised on the fourth day of prescription, but length can vary according to the department. Antifungal treatment is revised on the following day of prescription.

^c^ When administered intravenously.

**TABLE 2:** Characteristics of antimicrobial stewardship program (ASP) interventions in solid‐organ transplantation (SOT) recipients in University Hospital “12 de Octubre” (HU12O) from 2017 to 2021

**Variable**	**Total (*n* = 137 recipients)**
Type of SOT recipient (*n* [%])	
Lung transplant	68 (49.6)
Kidney transplant	51 (37.2)
Liver transplant	8 (5.8)
Heart transplant	8 (5.8)
Kidney‐Pancreas transplant	1 (0.7)
Multivisceral transplant	1 (0.7)
Treatment prescribed (*n* [%])^a^	
Antibiotic	116 (84.7)
Antifungal	22 (16.0)
Type of recommendation given (*n* [%])	
Withdrawal of antimicrobial treatment	71 (51.8)
Change to a better therapeutic regimen	36 (26.3)
Change from IV to PO route	9 (6.6)
Accept treatment prescribed^b^	21 (15.3)
Acceptance of the given recommendation (%)	92.4

^a^ One recipient was concomitantly receiving an antibiotic and an antifungal drug.

^b^ In 21 recipients, after reviewing the case with the attending physician, it was consensually decided to maintain the prescribed antimicrobial treatment.

IV: intravenous; PO: oral; SOT: solid organ transplantation.

Spain has various regional ASP, such as VINCat, a program of the Catalan Health Service that established a unified surveillance system for nosocomial infections and antimicrobial control in hospitals in Catalonia, and PIRASOA, a comprehensive program responsible for the prevention and control of healthcare‐associated infections, and the appropriate use of antimicrobials in the Andalusian Health Service. Although both programs publish annually their activities and results, data concerning SOT patients is still lacking.^24^, ^25^

### 1.3 Future directions

Spanish hospitals should make an effort in implementing ASP aimed at optimizing the antimicrobial treatment administered to SOT recipients. These programs should be patient‐centered and multidisciplinary, involving transplant infectious diseases (TID) physicians specialized in immunosuppressed patients and in antimicrobial stewardship, clinicians who attend SOT patients, microbiologists and pharmacists with expertise in immunocompromised patients,^26^ through the integration of clinical interventions and the microbiology laboratory (coupling antimicrobial and diagnostic stewardship are essential when treating SOT recipients). The ASP team would favor antimicrobial optimization, advise on the use of appropriate diagnostic tests (e.g., cost‐effective and rapid diagnostic assays), and also reduce the time required for communicating the microbiological results, thus improving the patient´s outcome.

The ASP team should also be responsible for creating organ‐specific clinical practice guidelines for infectious syndromes in SOT. These must be institution‐specific and adapted to the local epidemiology, as well as easily accessed by other clinicians.

Spanish centers should also implement automated alerts that notify the ASP team and TID specialists of particular antimicrobials or microorganisms isolated in SOT recipients, and request consultation and follow‐up. Such a strategy has proven to be an opportunity for improving antimicrobial treatment and the outcome for SOT recipients.^27^ Artificial intelligence pilot projects, such as the Wise Antimicrobial Stewardship Program Support System or the Development of an Expert System that Supports the Clinical Decision in the Management of Patients with Bacterial Infection (Roche) are currently underway in Spain and could be a useful tool in the future if applied to SOT.

Finally, ASP already taking place in Spanish medical centers that perform SOT should report their experience in this population. Until SOT‐specific ASP monitoring measurements are better defined, these programs should employ the quality metrics and process and outcome indicators routinely used to determine the impact of an ASP.^14^

## 2 CONCLUSION

SOT remains the best therapeutic option for end‐stage organ disease. Unfortunately, SOT recipients are disproportionately affected by antimicrobial adverse events, such as CDI, and infections produced by MDR microorganisms, which have a negative impact on the allograft function and the patient´s morbidity and mortality. The positive impact of an ASP coupled with a HAIC program in SOT has been recognized and should be applied in Spanish centers that perform transplantation. Automated alerts have also proven to be an opportunity for improving antimicrobial treatment and the outcome of SOT recipients. Finally, Spanish centers which already have an ASP implemented should report their results in SOT. Understanding the efficacy and safety of the Spanish ASP´s intervention in the SOT population is essential and deserves further study.

## CONFLICT OF INTEREST

The authors declare no conflict of interest.
